# Prevalence and Risk Factors of Postpartum Depression and Its Impact on Neonatal Health: A Cross-Sectional Study in Riyadh, Saudi Arabia

**DOI:** 10.7759/cureus.82819

**Published:** 2025-04-23

**Authors:** Baraa A Mazi, Eyesha Junaidallah, Nawaf N Alotaibi, Abdulaziz A Alharbi, Dania AlJaroudi, Ahmed Saleh, Fatimah S Alshahrani, Maryam A Althamin

**Affiliations:** 1 Mental Health, King Fahad Medical City, Riyadh, SAU; 2 Medicine, Almaarefa University, Riyadh, SAU; 3 Collage of Medicine, King Faisal University, Al-Ahsa, SAU; 4 Laboratory, King Fahad Medical City, Hail, SAU; 5 Reproductive Endocrine and Infertility Medicine Department, King Fahad Medical City, Riyadh, SAU; 6 Research Center, King Fahad Medical City, Riyadh, SAU; 7 Psychiatry, King Fahad Medical City, Riyadh, SAU; 8 Medicine and Surgery, Almaarefa University, Riyadh, SAU

**Keywords:** depression, newborn, outcomes, postpartum, prevalence

## Abstract

Objective

Postpartum depression (PPD) is a common yet often underdiagnosed mental health condition that can significantly affect maternal well-being and neonatal outcomes. Despite growing awareness, PPD remains insufficiently studied within specific cultural contexts, such as Saudi Arabia, where social and familial expectations may influence maternal mental health. This study aimed to assess the prevalence of PPD in Saudi women and investigate its association with neonatal health outcomes and maternal risk factors.

Methods

A retrospective cross-sectional study was conducted at the Psychiatry Department of King Fahad Medical City, Riyadh, Saudi Arabia, between 2019 and 2022. Medical records of 200 postpartum women were screened, and 56 eligible patients diagnosed with PPD were included. Diagnosis of PPD was based on clinical evaluation supported by the Edinburgh Postnatal Depression Scale (EPDS), a validated screening tool routinely used in the department. Data were collected using a structured review form covering demographic, clinical, and neonatal variables. Descriptive analysis was performed using the Statistical Package for the Social Sciences (SPSS). Odds ratios (OR) and 95% confidence intervals (CI) were calculated to assess the strength of association between PPD and potential risk factors.

Results

In this study, 56 Saudi Muslim women were investigated with a mean age of 35 years. The majority were married, 54 (92.9%). Regarding consanguinity, 25 (44.6%) had consanguineous marriages, 39 (69.6%) had planned pregnancies, and 28 (50%) females were suffering from different health conditions. Regarding symptom onset, 33 (58.9%) started to feel it after birth, and 23 (41.1%) had maternal complications. Most mothers were diagnosed with PPD after the birth of male infants, and 33 (58.9%) of the mothers were diagnosed with depression with their first newborn baby. Furthermore, 39 children (69.6%) suffered from chronic disease.

Conclusion

This study underscores the complex interplay of clinical, psychosocial, and obstetric factors in the development of PPD among Saudi mothers. Significant associations were identified with primiparity, vaginal delivery, life stressors, and pregnancy-related difficulties, emphasizing the need for early recognition and targeted support. Conversely, factors such as infant gender, consanguinity, and neonatal health issues were not significant predictors. These findings highlight the importance of culturally sensitive postpartum screening strategies and reinforce the value of integrated mental health services within maternal care frameworks to promote early intervention and optimize outcomes for both mother and child.

## Introduction

Postpartum depression (PPD) is a significant mental health condition affecting 10-20% of new mothers after childbirth [1]. It typically manifests within 4-6 weeks following delivery [2]. Globally, PPD is one of the most prevalent forms of depression among women [3], and it often progresses to major depression if left unaddressed [4]. The worldwide prevalence of PPD among mothers varies widely, from as low as 0.5% to as high as 60.8% [5]. Specifically, in Riyadh, the prevalence of PPD has been reported to be 35% [6]. PPD symptoms include persistent tiredness, low mood, trouble sleeping and focusing, diminished appetite, frequent crying without a clear reason, social isolation, and feelings of guilt regarding one’s ability to care for a newborn [7]. The Edinburgh Postnatal Depression Scale (EPDS) is a commonly used tool for assessing PPD in research and has been widely reported in studies to date [8]. Cultural factors, such as satisfaction with the gender of a newborn, can influence a mother’s emotional well-being [9]. The historical and cultural significance of gender preference remains a topic of human interest, and advancements in technology now allow parents to select their child's gender [10]. In societies with specific cultural norms, such as Saudi Arabia, there was a need for the validation of PPD risk factors. The community in Saudi Arabia plays a vital role in supporting new mothers, as cultural and social structures emphasize collective well-being and family cohesion. Given their awareness of PPD and its contributing factors, community members can provide crucial emotional and practical support to new mothers, potentially aiding in early prevention and intervention efforts [11,12]. For cultural reasons, the gender of the newborn might put the mother at risk for PPD. According to the findings of two studies conducted in Iran and South Africa, there is a relation between infant gender and PPD [10,11]. Additionally, research from the Czech has indicated that male gender newborns are more likely than female gender to develop PPD, while research from Argentina has revealed that having a female newborn increases the likelihood of developing PPD [12-14]. For instance, this study aimed to evaluate clinical characteristics and associated risk factors for women with PPD.

## Materials and methods

Study design

This study was designed as a retrospective cross-sectional review conducted at the Psychiatry Department of King Fahad Medical City in Riyadh, Saudi Arabia. The data collection spanned from 2019 to 2022 and focused on postpartum women diagnosed with PPD.

Ethical considerations

Prior to data collection, ethical approval was obtained from the Institutional Review Board (IRB) of King Fahad Medical City under IRB log number (22-387). Patient confidentiality was strictly maintained throughout the study, and all data were anonymized before analysis.

Study criteria

Inclusion criteria included women diagnosed with PPD within one year of delivery, patients managed at King Fahad Medical City between 2019 and 2022, and those with complete medical records containing demographic and clinical data relevant to the study.

Exclusion criteria involved women diagnosed with postpartum blues or depression unrelated to the postpartum period, those with a prior history of major depressive disorder or other psychiatric illnesses, incomplete medical records or unclear diagnoses, and cases where depression developed more than one year postpartum.

Procedure

A total of 200 patient records were initially screened. A thorough review process was applied to identify eligible records, using the above criteria. After exclusions, 56 cases were finalized for inclusion. The selection process was performed manually by trained research personnel with oversight by mental health specialists.

Tools and assessments

Data were collected using a structured chart review form specifically designed for this study. The form included information on sociodemographic characteristics, obstetric history, delivery details, gender preference, neonatal outcomes, and psychological health. Diagnosis of PPD was based on both clinical documentation and use of the EPDS, a validated 10-item self-report questionnaire widely used for identifying mothers at risk for PPD. In this hospital, EPDS is routinely administered during postpartum psychiatric assessments, and a cutoff score of ≥13 was considered indicative of probable depression, consistent with international standards. The data collection form was reviewed by maternal mental health experts and was piloted on a subset of records for clarity and reliability.

Sample size calculation

This study utilized a total sample of 56 postpartum women selected from an initial cohort of 200 patient records. Since the study design was descriptive and exploratory in nature, no formal sample size calculation was performed. Instead, all eligible patients meeting the inclusion criteria within the review period were included.

Statistical analysis

The study employed descriptive statistics to summarize the data, including frequencies, percentages, means, and standard deviations, using the Statistical Package for the Social Sciences (SPSS), version 25. In addition to descriptive analysis, odds ratios (OR) and corresponding 95% confidence intervals (CI) were calculated to evaluate the strength of association between PPD and various sociodemographic, clinical, neonatal, and psychosocial variables. This approach enabled a clearer assessment of potential risk factors contributing to the development of PPD.

## Results

The sociodemographic profile of the 56 women diagnosed with PPD revealed a mean age of 35.91 years, ranging from 20 to 48 years. The vast majority of participants were married 52 (92.9%), while a small proportion were divorced 4 (7.1%). All participants were Saudi nationals and adhered to the Islamic faith, reflecting the cultural and religious homogeneity of the study population. These characteristics provide context for understanding the psychosocial and cultural dynamics potentially influencing postpartum mental health within this group (Table 1).

**Table 1:** Sociodemographic Characteristics of Women Diagnosed with Postpartum Depression (PPD) (n = 56). This table presents the demographic profile of the 56 women diagnosed with PPD, including age distribution, marital status, nationality, and religion. The data provide context for understanding the cultural and social environment of the study population.

Variables		Frequency (Percentage)
Age (Mean = 35.91 years)	Minimum	20
	Maximum	48
Marital status	Married	52 (92.9)
	Divorced	4 (7.1)
Nationality	Saudi	56 (100)
Religion	Islam	56 (100)

Among the 56 women diagnosed with PPD, 25 (44.6%) had consanguineous marriages, with an associated odds ratio of 1.21 (95% CI: 0.62-2.34), compared to 31 (55.4%) without consanguinity. Planned pregnancies were reported by 39 women (69.6%), with a minimal association with PPD (OR = 0.98; 95% CI: 0.48-2.00), while 17 (30.4%) had unplanned pregnancies. Maternal complications were present in 23 women (41.1%), with a slight increase in odds of PPD (OR = 1.29; 95% CI: 0.66-2.54), compared to 33 (58.9%) who had no complications. Only three women (5.4%) reported using medications during pregnancy, with an odds ratio of 1.08 (95% CI: 0.25-4.68), whereas 53 (94.6%) did not use any medications. A significant association was observed with the use of antidepressants after delivery; 35 women (62.5%) received antidepressants, with a notably high odds ratio of 6.67 (95% CI: 3.21-13.83), compared to 21 (37.5%) who did not. These findings emphasize the potential impact of consanguinity, maternal complications, and particularly postnatal antidepressant use on the development and management of PPD (Table 2).

**Table 2:** Association Between Pregnancy and Health-Related Factors With Postpartum Depression (PPD). This table summarizes the relationship between various antenatal and health-related variables, such as consanguinity, pregnancy planning, maternal complications, medication use during pregnancy, and postpartum antidepressant use, and the likelihood of developing PPD, using OR and 95% CI.

Variables		Frequency (Percentage)	Odds Ratio	95% Cl
Consanguinity status	Yes	25 (44.6)	1.21	0.62 – 2.34
	No	31 (55.4)		
Planned pregnancy	Yes	39 (69.6)	0.98	0.48 – 2.00
	No	17 (30.4)		
Maternal complication	Yes	23 (41.1)	1.29	0.66 – 2.54
	No	33 (58.9)		
Usage of medications during pregnancy	Yes	3 (5.4)	1.08	0.25 – 4.68
	No	53 (94.6)		
Usage of antidepressants after delivery	Yes	35 (62.5)	6.67	3.21 – 13.83
	No	21 (37.5)		

In relation to newborn characteristics and their association with PPD, 32 mothers (57.1%) were diagnosed with PPD following the birth of a male infant, compared to 24 (42.9%) who had female infants, with an odds ratio of 1.49 (95% CI: 0.63-2.71). A higher occurrence of PPD was noted among first-time mothers (primiparous), accounting for 33 cases (58.9%), with an odds ratio of 2.15 (95% CI: 1.11-4.19), indicating a significant association when compared to multiparous mothers (23 cases, 41.1%). Most mothers (54; 96.4%) reported that the gender of the baby matched their expectations, with a corresponding odds ratio of 1.81 (95% CI: 0.29-11.39), while only 2 (3.6%) experienced a mismatch. Regarding gestational age, 9 (16.1%) had preterm deliveries and 47 (83.9%) had full-term deliveries, with an odds ratio of 1.55 (95% CI: 0.63-3.75). In terms of delivery method, 23 women (41.1%) had vaginal deliveries and 33 (58.9%) underwent cesarean section; vaginal delivery was associated with a higher odds of PPD (OR = 2.15; 95% CI: 1.11-4.19). Delivery complications were reported in 12 cases (21.4%) versus 44 (78.6%) without complications, showing a minor association (OR = 1.09; 95% CI: 0.48-2.50). Finally, 17 infants (30.4%) had health problems, associated with a slightly increased odds of maternal PPD (OR = 1.31; 95% CI: 0.63-2.71), compared to 39 (69.6%) who were healthy (Table 3).

**Table 3:** Association Between Newborn-Related Characteristics and Postpartum Depression (PPD). This table explores the influence of infant-related factors such as gender, parity, gestational age, mode of delivery, delivery complications, and neonatal health status on the occurrence of PPD, expressed in odds ratios (OR) and 95% confidence intervals (CI).

Characteristics	Category	Frequency (Percentage)	Odds Ratio	95% Cl
Newborns’ gender while diagnosed with PPD	Male	32 (57.1)	1.49	0.63 – 2.71
	Female	24 (42.9)		
Birth order while diagnosed with PPD	Primiparous	33 (58.9)	2.15	1.11 – 4.19
	Multiparous	23 (41.1)		
Gender remained the same after birth	Yes	54 (96.4)	1.81	0.29 – 11.39
	No	2 (3.6)		
Pregnancy term	Preterm (>38 weeks)	9 (16.1)	1.55	0.63 – 3.75
	Full term	47 (83.9)		
Delivery route	Vaginal	23 (41.1)	2.15	1.11 – 4.19
	C-section	33 (58.9)		
Delivery complication	Yes	12 (21.4)	1.09	0.48 – 2.50
	No	44 (78.6)		
Babies with health problems	Yes	17 (30.4)	1.31	0.63 – 2.71
	No	39 (69.6)		

Among the mothers diagnosed with PPD, 37 (66.1%) reported satisfaction with their baby’s gender, while 19 (33.9%) were dissatisfied. The odds ratio for developing PPD among those satisfied was 1.30 (95% CI: 0.83-3.12), indicating no strong association. Regarding spousal satisfaction, 40 women (71.4%) reported that their husbands were satisfied with the baby, with an odds ratio of 1.35 (95% CI: 0.66-2.74), compared to 16 (28.6%) who reported dissatisfaction. A significant association was found with exposure to stressful life events; 49 mothers (87.5%) reported such events, showing a markedly elevated odds ratio of 7.00 (95% CI: 2.89-16.94), in contrast to 7 (12.5%) who did not experience stressors. Fear of failure regarding motherhood was reported by 29 women (51.8%), with an odds ratio of 1.61 (95% CI: 0.83-3.12), while 27 (48.2%) denied such fears (Table 4).

**Table 4:** Influence of Postnatal Social Factors on the Risk of Postpartum Depression (PPD). This table evaluates psychosocial variables, including maternal and paternal satisfaction with the baby’s gender, exposure to stressful life events, and maternal fear of failure, and their association with PPD among the study participants using calculated odds ratios (OR) and 95% confidence intervals (CI).

Variables		Frequency (Percentage)	Odds Ratio	95% Cl
Satisfaction with baby gender	Yes	37 (66.1)	1.30	0.83 – 3.12
	No	19 (33.9)		
Satisfaction of the husband	Yes	40 (71.4)	1.35	0.66 – 2.74
	No	16 (28.6)		
Stressful life events	Yes	49 (87.5)	7.00	2.89 – 16.94
	No	7 (12.5)		
Fear of failure regarding motherhood	Yes	29 (51.8)	1.61	0.83 – 3.12
	No	27 (48.2)		

Out of the 56 women with PPD, 43 (76.8%) reported experiencing difficulties during pregnancy, which was significantly associated with PPD (OR = 4.96; 95% CI: 2.37-10.38), compared to 13 women (23.2%) who reported no such difficulties. Among those who experienced pregnancy-related challenges (n = 43), psychological difficulties were the most commonly reported, affecting 26 women (60.5%) with an odds ratio of 1.76 (95% CI: 0.75-4.14). Physical difficulties were noted in 14 women (32.5%) but showed a weaker association with PPD (OR = 0.90; 95% CI: 0.37-2.20). Financial difficulties were reported by 11 women (25.6%), with an odds ratio of 0.89 (95% CI: 0.34-2.31), while social difficulties affected 12 women (27.9%), showing a slightly elevated but non-significant association (OR = 1.13; 95% CI: 0.43-2.93) (Table 5).

**Table 5:** Association of Pregnancy-Related Difficulties With Postpartum Depression (PPD). This table assesses the overall impact of pregnancy-related difficulties categorized into physical, psychological, financial, and social domains on the risk of developing PPD, using calculated odds ratio and 95% confidence interval.

Variables		Frequency (Percentage)	Odds Ratio	95% Cl
Difficulties during pregnancy	Yes	43 (76.8%)	4.96	2.37 – 10.38
	No	13 (23.2%)		
Type of Difficulties (n=43)				
Physical difficulties	Yes	14 (32.5)	0.90	0.37 – 2.20
Psychological difficulties	Yes	26 (60.5)	1.76	0.75 – 4.14
Financial difficulties	Yes	11 (25.6)	0.89	0.34 – 2.31
Social difficulties	Yes	12 (27.9)	1.13	0.43 – 2.93

## Discussion

The mean maternal age was 35.9 years, and while we did not find age to be a significant factor, this is consistent with some reports that PPD can affect women across a wide age range [4]. Most participants (92.9%) were married, leaving too few non-married cases to evaluate marital status as a risk factor; however, other studies have noted that lacking a partner or being single can increase PPD risk [12]. For example, a large Czech study found that mothers living without a partner had higher odds of depression postpartum, underscoring the protective influence of marital support. Our cohort’s unique cultural factor - consanguineous marriage (44.6% of cases) - showed a small, non-significant increase in PPD odds (OR = 1.21, CI: 0.62-2.34). We did not find literature explicitly linking consanguinity to PPD; the lack of association in our setting suggests that being in a cousin marriage per se neither heightened nor protected against PPD. Overall, the absence of significant sociodemographic predictors in our study may reflect the relatively uniform profile of our participants (all Saudi, all Muslim, predominantly married). It also aligns with findings that PPD risk is not confined to particular ages or basic demographics [1,12]. Nonetheless, it is important to note that social context and support (rather than marital status alone) play a critical role, as discussed under psychosocial factors. Several pregnancy and health-related factors were evaluated, with mixed outcomes. Notably, whether the pregnancy was planned did not substantially affect PPD occurrence in our sample (OR = 0.98, CI: 0.48-2.00). About 30% of depressed mothers had unplanned pregnancies, but this proportion was similar among those with planned pregnancies. Our finding contrasts with some studies that report unwanted or unplanned pregnancies as a significant risk factor for PPD. For instance, an Iranian study observed higher depression rates in mothers with unwanted pregnancies [10], and a Czech cohort found that “unintentional pregnancy” was associated with more depressive symptoms in univariate analysis [12]. The discrepancy could be due to cultural differences in the impact of unplanned pregnancy; in our Saudi context, even if a pregnancy is unplanned, strong family support or acceptance might buffer the psychological impact, whereas in other settings, an unintended pregnancy may induce greater stress or socioeconomic strain.

Maternal health complications during pregnancy or delivery showed only a modest and non-significant relation with PPD (OR = 1.29, CI: 0.66-2.54 for mothers with any complication vs. none). Roughly 41% of PPD cases had reported complications (such as gestational diabetes or hypertension), but this rate did not markedly exceed that in non-PPD women. While our data did not reach significance, it is plausible that health complications add to stress and could contribute to PPD risk. Some prior research has linked difficult pregnancies or obstetric complications to postpartum depressive symptoms [10], though results are inconsistent. Our study’s small sample may limit the power to detect these effects. Similarly, use of medications for medical conditions during pregnancy was rare (5.4%) and not significantly associated with PPD (OR ≈ 1.08). This suggests that chronic maternal physical illness requiring medication was not a primary driver of PPD in our cohort, or that those few cases did not differ from others in depression risk. One notable finding was that mothers who experienced overall difficulties during pregnancy had a substantially higher likelihood of PPD. Experiencing any significant difficulty (whether physical, emotional, financial, or social) was associated with about a five-fold increase in odds of PPD (OR = 4.96, CI: 2.37-10.38). This aligns with the understanding that the cumulative stress of pregnancy-related challenges predisposes women to postpartum depression. Consistent with our result, Fiala et al [12] identified psychosocial stressors during pregnancy as one of the strongest predictors of PPD in their large sample. In our data, 76.8% of depressed mothers reported some form of serious difficulty while pregnant. When broken down by type, however, none of the specific categories (physical, psychological, financial, or social difficulties) individually showed a statistically significant effect on PPD. For example, psychological difficulties (such as high anxiety or emotional stress during pregnancy) had an OR of 1.76 (95% CI: 0.75-4.14) for subsequent PPD, suggesting a possible trend, but our sample size was too limited to confirm it. Financial difficulties during pregnancy did not show any increase in PPD risk in our group (OR = 0.89, CI: 0.34-2.31), whereas a study in the Czech Republic found that having financial savings (an indicator of economic stability) was protective against PPD. This might indicate that, in our context, financial stress was less acute or was mitigated by extended family support networks. Overall, the presence of any hardship during pregnancy appears more consequential than the category of hardship, pointing to a cumulative stress effect. This underscores that clinicians should monitor pregnant women facing multiple stresses or complications, as they may be at higher risk for PPD. Lastly, it is worth mentioning that over 60% of the mothers with PPD in our study required antidepressant treatment postpartum, a rate significantly higher than those who did not develop PPD (OR = 6.67, CI: 3.21-13.83 for receiving postpartum antidepressants).

While this is not a risk factor for PPD (rather, it is a consequence of PPD diagnosis and management), it does indicate the clinical severity of our cases. Many women needed pharmacotherapy, reflecting that their depression was recognized and treated. The high OR here mainly signifies that PPD cases were correctly identified and managed with medication; it also highlights the importance of postpartum follow-up and access to psychiatric care in mitigating PPD’s impact. We explored whether characteristics of the newborn or the birth event were associated with maternal depression. One aspect of interest was the baby’s sex. In our sample, a higher proportion of women who became depressed had given birth to male infants (57.1% of PPD cases had a boy) compared to female (42.9%), corresponding to an OR of 1.49 (CI: 0.63-2.71) for having a male baby. This OR was not statistically significant, indicating that infant gender by itself was not a strong predictor of PPD in our study. The literature on infant sex and PPD is mixed and often culturally dependent. Some studies have found that not having the culturally preferred gender can affect the mother’s mood. For example, a study among adolescent mothers in Indonesia reported that gender preference contributed to higher PPD levels, presumably when the baby’s sex did not match family expectations [14]. In contrast, research in Saudi Arabia generally shows no significant link between a baby’s sex and PPD, similar to our findings. Two studies from Riyadh found that PPD rates did not differ between mothers of boys and girls [15,16]. Likewise, an Iranian study reported no association between infant gender and maternal depression [10]. Interestingly, in other cultural contexts the influence of infant sex has been observed in opposite directions: a large Czech study noted that having a male infant was associated with lower PPD risk (suggesting mothers of girls were more prone to depression [12], whereas a study in Argentina found that mothers of female babies had higher depression scores [13]. These inconsistent findings likely reflect differing cultural values and expectations - in some societies, a male child is more desired, potentially protecting the mother from depressive feelings if a son is born, whereas in others, or under different personal circumstances, a daughter might be preferred. In our cohort, nearly all women (96.4%) stated that the baby’s sex met their prior expectation or preference, which may explain why infant gender did not play a major role in PPD. There was virtually no “gender disappointment” in our sample; only two mothers (3.6%) had an unexpected baby sex, and this small subgroup was insufficient to draw conclusions (OR = 1.81, very wide CI: 0.29-11.39). Overall, our findings and the regional literature indicate that infant sex alone is not a consistent predictor of PPD in Saudi Arabia [15,16], unlike in some settings where strong gender preference norms exist. Cultural context is likely key - in Saudi culture, both male and female newborns are generally welcomed, and any subtle preferences rarely escalate to clinical depression in the mother. First-time motherhood (primiparity) emerged as a significant factor in our analysis. Mothers experiencing their first childbirth had more than double the odds of PPD compared to multiparous mothers (OR = 2.15, CI: 1.11-4.19). In fact, 58.9% of our PPD cases were first-time mothers. This suggests that the transition to motherhood for the first time - with its new responsibilities, uncertainty, and potential anxiety - may make women more vulnerable to depression.

Our result is in line with the findings of Alzahrani [6], who reported that primiparity was a notable risk factor for postnatal depression among Saudi women. It is plausible that in our context, women with no prior parenting experience may feel less prepared and more overwhelmed, which can precipitate depressive symptoms. International studies have also discussed the vulnerability of first-time mothers. For instance, some authors have noted that a lack of familiarity with infant care and the stress of a new maternal role can increase the risk of PPD [12]. Interestingly, not all research agrees on this point: the Czech ELSPAC study by Fiala et al. actually found that primiparous mothers had a lower risk of PPD at 6 months postpartum than multiparas. The authors suggested that experienced mothers (with multiple children) might face more cumulative stress or have less support postpartum, whereas a first baby often mobilizes additional family support [12]. In our region, however, it appears the challenges of new motherhood outweigh any additional support primiparas might receive. The conflicting evidence highlights that the impact of parity on PPD can vary - possibly first-time mothers are more at risk in some contexts (as in ours and Alzahrani’s findings), while in others, mothers of multiple children might struggle more. Cultural expectations and family structure could explain this difference. In Saudi Arabia, extended families often assist with the first baby, but the internal pressure a woman puts on herself to be a “good mother” for the first time may still be intense, contributing to PPD. Clinicians should thus pay special attention to primiparous women, offering extra support and education to help mitigate their risk of depression. We also considered neonatal health outcomes as potential influences on PPD. A considerable subset of our depressed mothers had infants with health problems (30.4% reported that their baby had a health issue, such as a chronic illness or significant neonatal condition). We found a trend toward higher PPD odds when the infant had health problems (OR = 1.31), but this was not statistically significant (CI: 0.63-2.71). The majority (70%) of PPD cases were mothers of healthy babies, suggesting that infant illness alone was not a decisive factor in our study. Still, caring for an unwell infant can be very stressful for mothers, and other research has pointed to a link between neonatal complications (like prematurity or NICU admission) and maternal depression. For example, some studies report that mothers of very preterm or medically fragile infants experience higher levels of depressive symptoms due to worry and caregiving strain (though such findings are not unanimous). In Fiala et al.’s large study, having a newborn who required intensive care was examined, and ultimately, no clear association with PPD was found, which is in line with our result. It may be that the influence of infant health on maternal mood is significant in acute situations (e.g., immediately postpartum or in cases of severe infant illness) but less detectable by the time PPD is diagnosed, especially if the sample size is limited. Additionally, in our context, many infant health issues may be supported by family members or the healthcare system, potentially buffering the impact on the mother’s mental health. Overall, while we did not find a significant relationship, it remains important to monitor mothers of infants with health problems, as they could have elevated stress that might contribute to depression in some cases. Other birth-related factors were evaluated, including gestational age and mode of delivery. We observed a higher proportion of PPD among mothers who had preterm births (<38 weeks, 16.1% of cases) compared to the general population rate of preterm deliveries, but statistically this did not translate into a significant risk increase (OR = 1.55, 95% CI: 0.63-3.75 for preterm vs. full-term). This is consistent with Fiala et al.’s findings that gestational maturity of the baby was not a significant predictor of PPD [12].

Although extreme prematurity can induce emotional distress, many of our “preterm” cases may have been only mildly preterm, and their infants possibly did well, thereby not strongly affecting the mother’s mood by the time of assessment. An intriguing result in our study was the association with delivery method. Contrary to some expectations, mothers who had a normal vaginal delivery showed higher odds of PPD compared to those who delivered by cesarean section (OR = 2.15, CI: 1.11-4.19). In our data, 41.1% of PPD cases had vaginal births, whereas 58.9% had C-sections, and vaginal delivery emerged as a significant risk factor. This finding appears counterintuitive, as one might assume surgical delivery could heighten depression risk due to pain, longer recovery, or an unplanned operative birth experience. Indeed, many studies have reported C-section as a risk factor for postpartum depression. For example, in a Chinese cohort, Xie et al. found the PPD rate to be about twice as high in women who had cesarean deliveries (21.7%) compared to those who delivered vaginally (10.9%) [17]. Their analysis indicated that cesarean section was associated with significantly increased odds of PPD. A 2017 meta-analysis by Xu et al. also concluded that cesarean delivery is linked with elevated risk of PPD across diverse populations [18]. On the other hand, some studies (including one in Iran) found no significant difference in PPD rates between vaginal and C-section deliveries [10]. Our result aligns more with the findings of Almutairi et al. [16] in Riyadh, who reported that women who underwent vaginal deliveries had higher depression scores postpartum than those with C-sections. In Almutairi’s study, after controlling for confounders, normal vaginal delivery was associated with increased depressive symptom levels, whereas cesarean delivery (especially if elective) did not show that increase. The reasons behind this counterintuitive trend in our setting are speculative but may relate to the circumstances surrounding each delivery mode. It is possible that some of the vaginal deliveries in our sample were particularly traumatic or painful, leading to physical and psychological stress that contributed to PPD. For instance, an unexpected difficult labor, severe perineal tear, or inadequate pain management could leave a new mother distressed, whereas a planned C-section might have been a more controlled experience. Additionally, women who required C-sections in our sample might have received more attention and postpartum care due to the surgical recovery, including support from family during the longer healing period, which could mitigate depression. Cultural attitudes might also play a role; if a vaginal birth is expected as the norm, a mother who has a tough vaginal delivery may still feel pressure to recover quickly and care for the newborn, potentially feeling overwhelmed. In contrast, a mother who had a C-section might feel more justified in seeking help and rest, thereby reducing her risk of depression. Given that our finding diverges from much of the international literature [17,18], it should be interpreted with caution. It underlines that risk factors for PPD are context-dependent. Healthcare providers in our region should be aware that even women with “routine” vaginal births may need screening and support for PPD. Meanwhile, the absence of a higher risk in post-cesarean mothers in our study does not negate the importance of monitoring that group, as other studies have warned.

Further research with larger samples is needed to clarify how and why delivery mode interacts with maternal mental health in different cultural and clinical settings. Psychosocial stressors and support systems were among the most influential factors associated with PPD in our study, which is consistent with a broad body of evidence. Nearly 88% of the mothers with PPD reported experiencing one or more stressful life events around the time of pregnancy or postpartum. These could include events like family conflicts, financial crises, loss of a loved one, or other significant life changes. We found that exposure to stressful life events was strongly linked to PPD (OR = 7.00, 95% CI: 2.89-16.94). In fact, this was one of the strongest associations observed, indicating that mothers who had major stressors were many times more likely to develop PPD than those without such stress. This finding reinforces what has been “extensively documented” in the literature: stressful events can precipitate or exacerbate depression [19,20]. For example, Assari and Lankarani [20] showed that cumulative life stressors were associated with higher long-term depression risk in women. In the postpartum context, Al Nasr et al. [15] identified stressful life events as a significant predictor of PPD in Riyadh (OR ~2.7 in their regression model), and our result underscores the same relationship, albeit with an even higher OR, possibly due to our focused PPD sample. The broad confidence interval in our estimate suggests some uncertainty, but the lower bound of ~2.9 still indicates at least a threefold increase in odds, which is clinically meaningful. The clear takeaway is that stress matters: women who endure serious life difficulties around childbirth are at a much higher risk for PPD. Culturally, Saudi women often live within extended families, so a “stressful life event” affecting the family (such as a death or a marital conflict) can directly impact the new mother’s emotional well-being. It is crucial for healthcare providers to screen postpartum women for recent life stressors - those who have faced significant adversity may need proactive mental health support or counseling to prevent PPD or lessen its severity [20]. Our findings contribute to the evidence that mitigating life stress (where possible) and offering resources during crises could reduce PPD incidence. In terms of social support, our study looked at a few proxy measures related to the family’s reaction to the baby. We found that maternal satisfaction with the baby’s gender and the husband’s satisfaction with the baby were not significantly associated with PPD. Two-thirds of depressed mothers were personally happy with their baby’s sex (66.1% were satisfied, vs. 33.9% unsatisfied), and those who were unsatisfied did not have significantly different odds of PPD (OR = 1.30, CI: 0.83-3.12). Similarly, 71.4% of PPD mothers reported that their husband was happy with the baby, and lack of the husband’s satisfaction was not a strong predictor (OR = 1.35, CI: 0.66-2.74). These findings suggest that gender disappointment was not a major driver of depression in our sample - a conclusion also reflected in our earlier discussion on infant sex. Most families in our study accepted the newborn’s gender, so support from the spouse or family did not hinge on the baby being a boy or girl. However, “husband’s satisfaction” in our data is a limited indicator of support. It specifically relates to contentment with the child (likely tied to gender), rather than the overall emotional or practical support the husband provides to the mother. When we examine the broader concept of partner support, the literature evidence strongly indicates that poor support from the father of the baby increases PPD risk. Al Nasr et al. [15] found that having an unsupportive spouse was a significant predictor of PPD (with an adjusted OR around 4.5 in their study). In our cohort, we did not directly measure general partner support with a scale, but we infer its importance from both the literature and related variables. For instance, Moradi et al. [10] documented a significant relationship between low spousal support and higher PPD levels among Iranian mothers, and Almutairi et al. [16] reported that lower partner support was associated with more severe depressive symptoms in Saudi women. In addition, a recent study in Qassim, Saudi Arabia, noted that women who lacked support from their husbands (or had marital conflicts) were more likely to screen positive for PPD [5]. These findings are congruent with the general understanding that an involved, supportive partner can buffer the stresses of new motherhood, whereas a disengaged or critical partner can worsen them. Our results did show a slight trend that if the husband was dissatisfied (perhaps indicating some conflict or disappointment), the odds of PPD were higher (OR >1), though not significantly so. It is likely that our proxy measure did not capture the nuanced aspects of spousal support. Considering both our data and others’, it is clear that psychosocial support from the partner and family is protective. Indeed, existing evidence emphasizes that strong family support can significantly reduce the incidence of PPD [8,16].

In practice, this means that ensuring the mother has adequate help, understanding, and encouragement from her spouse and relatives may lower her risk of developing depression. Culturally, Saudi women often have their mothers, sisters, or in-laws involved after childbirth; such support could be one reason why factors like marital status or parity sometimes show different effects here than in Western contexts. Our findings reinforce the notion that social support interventions (like educating spouses about PPD and involving them in postpartum care) could be beneficial [10]. Even though “husband’s satisfaction with the baby” wasn’t a determinant of PPD per se, the husband’s support for the mother likely is a factor, indirectly evidenced by the high prevalence of PPD when stressors were present and the protective trends seen when no conflict was reported [5]. Another psychosocial variable we assessed was the mother’s fear of failing in her maternal role. Just over half (51.8%) of the women with PPD acknowledged feeling a fear that they might fail as a mother. We found the odds of PPD were modestly higher in those who had such fears (OR = 1.61), but this did not reach statistical significance (CI: 0.83-3.12). This suggests that while self-doubt about motherhood is common, it alone did not distinguish who became depressed in our study. It is possible that this fear is as much a symptom or correlate of depression as it is a cause - for example, a mother with PPD may experience more feelings of inadequacy. It’s also possible that many mothers without depression still have occasional worries about their new role, meaning this sentiment is not specific enough to predict PPD on its own. There is limited direct research on “fear of motherhood failure” as a quantified risk factor, but it relates to the concept of self-esteem and efficacy in the maternal role. Low self-esteem and high anxiety have been linked to PPD in prior studies[5,16], so one might expect that severe fear of not being a good mother could contribute to PPD. Our findings imply that such fears, when not coupled with other factors, were not sufficient to trigger depression in most cases. Mothers who did not report this fear (48.2% of our PPD cases) still developed PPD due to other stressors. In the context of Saudi culture, first-time mothers often receive guidance from experienced family members, which might alleviate or invalidate their personal fears about parenting inadequacy. Thus, while clinicians should listen for expressions of overwhelm or self-doubt (as they can be early signs of distress), interventions might be more effectively targeted at external stress and strengthening support, rather than solely addressing a mother’s inner worry about failure. In summary, the psychosocial domain in our study clearly shows that external stresses and supports are pivotal. High stress amplifies the risk of PPD markedly, whereas strong support systems (especially partner support) are likely protective, even though our proxy measures did not capture support effects quantitatively. These findings are strongly aligned with the literature: for example, Al Nasr et al. [15] and others identify stressful life events and lack of support as key predictors of PPD, whereas abundant social support is consistently associated with lower PPD rates [5,16].

Culturally contextual factors - such as extended family involvement and societal expectations - can modulate these effects, but the core principle remains that helping mothers manage stress and bolstering their support network should be central to PPD prevention strategies. Our results, together with prior studies, highlight the need for healthcare providers to screen for psychosocial stressors and ensure new mothers are not left isolated. Interventions like counseling for those with recent life events, and educating spouses/families about the importance of their support, could significantly improve maternal mental health outcomes [16]. Our cross-sectional findings resonate with much of the existing literature on PPD risk factors, while also reflecting the specific cultural context of our population. Sociodemographic variables (age, marital status, consanguinity) showed no significant impact on PPD in our cohort, consistent with some international observations that PPD spans across ages and social strata [12]. Clinical and pregnancy-related factors such as unplanned pregnancy and obstetric complications were not major determinants of PPD in our sample, although experiencing any significant difficulties during pregnancy was a clear risk, reinforcing the connection between prenatal stress and postpartum depression. Newborn-related factors revealed that primiparous women were at higher risk for PPD [6], aligning with the notion that first-time motherhood can be challenging, though parity effects can differ by context [12]. Infant sex was not a decisive factor for us, which is in line with other studies from the region [15] and reflects relatively neutral gender attitudes in our setting, despite contrary findings in cultures with strong gender preference [13,14]. Perhaps most importantly, psychosocial and support factors played a central role: mothers who endured stressful life events had a much greater likelihood of PPD, whereas those with strong spousal and family support tended to fare better, echoing extensive evidence on stress and support as universal factors in postpartum mental health [15,16,19]. The consistency and inconsistencies between our findings and others’ underline how PPD is a multi-factorial condition with core risk factors (like stress and lack of support) that transcend settings, and other factors whose effects may be context-dependent (like infant gender or parity). Understanding these nuances - including possible cultural reasons for differences - is important for developing targeted interventions. For instance, in a culture where family support is generally high, extra focus might be needed on identifying those few women who fall through the cracks of support, or those encountering severe external stresses. Our findings underscore the need for comprehensive postpartum care that includes psychosocial screening. Clinicians should interpret odds ratios with their confidence intervals in mind: an OR significantly above 1 (with CI not crossing 1) for factors like primiparity or life stress indicates a reliable association that can inform risk assessment, while an OR around 1 or with a wide CI (as with consanguinity or fear of failure) suggests no effect or an imprecise estimate that warrants further investigation. By comparing and contextualizing these results with prior studies, we reinforce the evidence base for what increases the risk of PPD and what might protect against it. This scholarly understanding ultimately aims to improve early identification of at-risk mothers and to inform culturally appropriate support strategies, thereby improving maternal and neonatal outcomes in the postpartum period [16].

## Conclusions

The findings of this study provide critical insight into the specific maternal and psychosocial risk factors associated with PPD within the Saudi context. While traditional demographic variables such as age, consanguinity, and infant gender showed limited predictive value, the study identified primiparity, vaginal delivery, stressful life events, and antenatal difficulties as significant contributors to PPD. These results underscore the need to shift postpartum mental health strategies from demographic screening toward a more holistic assessment of psychosocial stressors and reproductive experiences. Culturally tailored interventions that address psychological resilience, provide partner and family education, and enhance support during the perinatal period are essential. Strengthening multidisciplinary postpartum care pathways, with integrated mental health evaluation, may significantly reduce the burden of undiagnosed PPD and improve both maternal and neonatal outcomes in Saudi Arabia and similar settings.
